# A cytoplasmic escapee: desmin is going nuclear

**DOI:** 10.3906/biy-2107-54

**Published:** 2021-12-14

**Authors:** Ecem KURAL MANGIT, Niloufar BOUSTANABADIMARALAN DÜZ, Pervin DİNÇER

**Affiliations:** 1 Department of Medical Biology, Faculty of Medicine, Hacettepe University, Ankara Turkey; 2 Laboratory Animals Research and Application Centre, Hacettepe University, Ankara Turkey

**Keywords:** Cytoskeleton, intermediate filament, desmin, nucleus, nuclear localization signal, nuclear export signal

## Abstract

It has been a long time since researchers have focused on the cytoskeletal proteins’ unconventional functions in the nucleus. Subcellular localization of a protein not only affects its functions but also determines the accessibility for cellular processes. Desmin is a muscle-specific, cytoplasmic intermediate filament protein, the cytoplasmic roles of which are defined. Yet, there is some evidence pointing out nuclear functions for desmin. In silico and wet lab analysis shows that desmin can enter and function in the nucleus. Furthermore, the candidate nuclear partners of desmin support the notion that desmin can serve as a transcriptional regulator inside the nucleus. Uncovering the nuclear functions and partners of desmin will provide a new insight into the biological significance of desmin.

## 1. Introduction

Intermediate filaments (IFs) are a protein superfamily of 10-nm fibrous polymers in eukaryotes. Together with microtubules and microfilaments, IFs form the basic structure of the cytoskeleton. IF family is formed by a large (>70 proteins) and diverse group of proteins, which are expressed in a tissue type-specific manner (Hesse et al., 2001; Rogers et al., 2004, 2005; Oshima, 2007). Ifs are mainly take part in maintaining cell and tissue integrity, but, beyond the traditional functions, they play essential roles in organelle and protein distribution (Brunet et al., 2004; Toivola et al., 2005). According to Human Intermediate Filament Database (Szeverenyi et al., 2008), there are 119 diseases associated with mutations in IF proteins, which points out the importance of IFs in medicinal studies. 

The ‘’cytosolic’’ IF proteins are now starting to emerge as nuclear elements. Many different studies suggest that these proteins can localize and function in the nucleus and bring new insight into cellular events (Kumeta et al., 2012). 

This review focuses on the functions and candidate nuclear binding partners of one particular cytoplasmic protein: desmin. 

**Figure 1:**
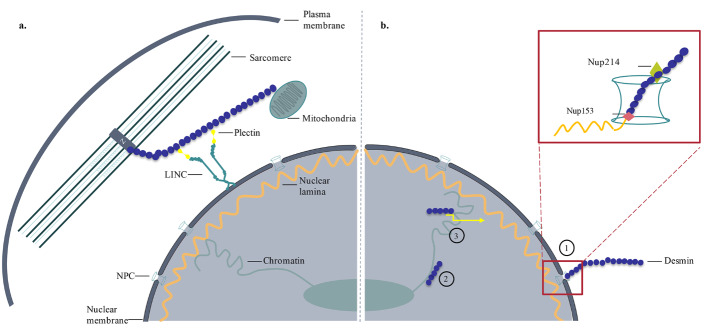
Functions of desmin in the cytoplasm (a) and the nucleus (b). (a) Desmin is mainly located at the Z-discs (Z) in the sarcoplasm and connects myofibrils to the nucleus and mitochondria (Lazarides, 1980; Fuchs and Weber, 1994). Through the Linker of Nucleoskeleton and Cytoskeleton Complex (LINC), desmin provides a mechanical link between the nucleus and the cytoskeleton (Stroud et al., 2014). (b) Inside the nucleus via lamin B association, desmin can provide static support to nucleoskeleton, involve in the nucleo-sarcoplasmic exchange ,or affect the DNA structure and function (1) (Lockard and Bloom, 1993). Desmin can modulate chromatin conformation (2) (Li et al., 1994) or regulate gene expression with other transcription factors (3). Desmin-lamin B interaction and their association with Nup153 and Nup214 are highlighted in the red rectangular area.

Desmin is a cytoplasmic muscle-specific type III IF. Through interaction with other cytoskeletal elements, desmin connects myofibrils to the nucleus, mitochondria, and sarcolemma and facilitates force transmission during muscle contraction (Lazarides, 1980; Fuchs and Weber, 1994) (Figure 1a). Desmin can act as a potential mechanosensor and transduce mechanical forces from the cytoplasm to the nucleus (Lockard and Bloom, 1993; Capetanaki et al., 2015). Mutations in the desmin gene (*DES*)* *cause skeletal and cardiac myopathies, collectively known as desminopathies.

## 2. Evidence of desmin localization in the nucleus

Except for type V IFs lamins, no IFs are expected to be localized in the nucleus. Yet, there are evidence from different studies implicating that the IFs other than lamins may localize in the nucleus. Desmin is one of the interesting examples. One of the oldest pieces of evidence about nuclear localization is the presence of desmin in the nucleus of BHK21 cells (Kamei, 1986). Not only it resides in the nucleus, but it has also been shown that desmin is a nucleic acid-binding protein in vitro (Traub and Shoeman, 1994; Tolstonog et al., 2000, Wang et al., 2001; Tolstonog et al., 2005). Furthermore, our studies have revealed that desmin can co-localize with lamin B at the nuclear periphery in the human skeletal muscle sections (Çetin et al., 2013). Finally, desmin has been found to be localized in the nucleus of differentiating embryonic stem cell-derived cardiac progenitor cells (Fuchs et al., 2016). Unfortunately, there is a relatively small body of literature that is concerned with the nuclear localization of desmin (Kamei, 1986; Traub and Shoeman, 1994; Hartig et al., 1998; Tolstonog et al., 2000, Wang et al., 2001; Tolstonog et al., 2005; Fuchs et al., 2016).

What can a cytoskeletal protein do in the nucleus?** **The former far-fetched idea of cytoskeletal proteins being in the nucleus is now beginning to settle, and it is not too absurd to assume that they can take on important tasks at both compartments. Nonerythroid α-spectrin, a structural protein, is required to recruit DNA repair proteins (Sridharan et al., 2003). Myosin VI (MVI), an actin-based motor protein, is associated with proteins involved in nuclear/ribosomal processes (Majewski et al., 2018). ). α-actinin, an actin cross-linking protein, is actively transported between nucleus and cytoplasm and interacts with transcriptional regulators (Kumeta et al., 2010). There are also examples of intermediate filament proteins taking part in the nucleus. Vimentin, a type III IF like desmin, is suggested to be a part of a chromatin-modifying complex (Hartig et al., 1998). Type I IF keratin, has an impact on a transcriptional regulator’s nuclear localization and function (Hobbs et al., 2015). There are many other examples of cytoskeletal proteins localize in the nucleus (For review: (Kumeta et al., 2012; Hobbs et al., 2016)). For IFs, it is not unusual to present in the nucleus considering they all originated from lamin-like predecessor (Weber et al., 1989). As a matter of fact, there is a belief that IFs might appear as nuclear-localized elements in primitive organisms (Peter and Stick, 2015). The distinguishing aspect of lamins from other IFs is that they have a nuclear localization signal (NLS) (Loewinger and McKeon, 1988) and a C-terminal CaaX-isoprenylation motif, which targets lamins to the nuclear envelope (Holtz et al., 1989; Peter and Stick, 2015). But are the lamins only IFs that have an NLS?

**Figure 2:**
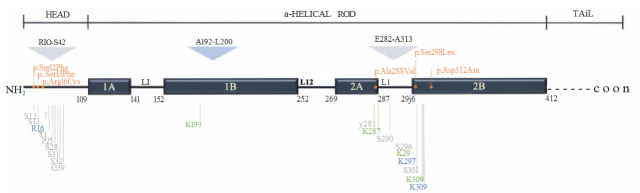
Schematic representation of the domains of desmin protein and the localization of NLSs, NES, PTMs, and mutations. Desmin protein comprises 470 amino acids and constitutes three regions, namely α- helical rod, head, and tail domain. The α- helical rod domain is interrupted by 3 linkers (L1, L12, and L2) that generate 4 coils (Coil 1A,1B,2A, and 2B). The head and rod domains are essential for IF assembly (Hnia et al., 2015; Fischer et al., 2021). The tail, on the other hand, seems to be involved in the organization of IF network (Hnia et al., 2015). The subscript numbers show at which amino acid the domains start and end. In the figure, light grey triangles represent the localization of NLSs on desmin protein, and the blue triangle represents NES. Gray lines at the bottom show phosphorylation, blue lines show acetylation and green lines show ubiquitination sites. Orange lines show the mutations within signal sequences on desmin protein. PTM data were obtained from PhosphoSitePlus Database (Hornbeck et al., 2015). Mutation data were obtained from The Human Intermediate Filament Database (Szeverenyi et al., 2008) A: Alanine; E: Glutamate; G: Glycine; K:Lysine; L: Leucine; P: Proline; R: Arginine;S: Serine; T:Threonine; Y: Tyrosine. Ala: Alanine; Arg: Arginin; Asn: Asparagine; Asp: Aspartic acid; Cys: Cysteine; Leu: Leucine; Phe: Phenylalanine; Ser: Serine; Val: Valine.

In the case of desmin, in silico analysis (La Cour et al., 2004; Kosugi et al., 2009) shows there are two potential NLS’s one starting at the Arginine 10 and ending at Serine 42 (R10-S42), and a second one starting at Glutamate 282 and ending at Alanine 313 (E282-A313) and one nuclear export signal (NES) starting at Alanine 192 and ending at Leucine 200 (A192-L200) (Figure 2). Localization of these potential signals on desmin is very interesting. One might envision that these signals become ‘accessible’ depending on the assembly or polymerization status of the protein since they are located on amino-terminal and rod domain, which are responsible for the assembly and coiled-coil polymerization of desmin, respectively (Costa et al., 2004; Hnia et al., 2015). The amino terminal of desmin is also a post-translational modification (PTM) site (Höllrigl et al., 2007; Mavroidis et al., 2008) (Figure 2) which indicates that the on/off status of the signal R10-S42 might be related to cell status since these PTMs are responsible for IF organization and structure through cell cycle and development (Mavroidis et al., 2008). The PTMs within NLSs and NES are illustrated in Figure 2. Among these PTMs, the amino-terminal phosphorylation might be specifically crucial for the subcellular localization of desmin. The phosphorylation status of the amino-terminal determines the polymerization-depolymerization status of desmin and other IFs (Geisler and Weber, 1988; Inagaki et al., 1988; Agnetti et al., 2021). According to a paper by Hobbs (2016), IFs destined to go to the nucleus — in this study, the referent IF is keratin — are expected to be small (newly synthesized or derived from existing IF network) and specified by PTMs or via interaction with other proteins (Hobbs et al., 2016). Considering the PTM sites along the amino-terminal NLS are responsible for the polymerization status of desmin; one can assume that NLS between R10-S42 might be activated during the polymerization-depolymerization cycle, and desmin filaments that are destined for nuclear transportation might be marked via phosphorylation. The investigation of the relationship between activation of NLS and phosphorylation would be interesting and valuable for understanding desmin localization. Other PTMs within the NLSs and NES are acetylation and ubiquitination. While ubiquitination is usually associated with proteasomal inhibition, acetylation is related to the protein solubility and insolubility depending on the location of this PTM (Snider and Omary, 2014). 

## 3. Binding partners and potential functions of desmin in the nucleus 

Fuchs (2016) discovered desmin occurs in the nuclei of differentiating cardiac progenitor cells and immature cardiomyocytes, presents in a transcription factor complex with nanog, brachyury, mesp1, and nkx2.5, and contributes to transcriptional regulation of cardiac-specific transcription factor *nkx2.5* during cardiomyogenesis (Fuchs et al., 2016). This study does not only pointing out that desmin can localize in the nuclei of cardiomyocytes but also presents evidence pointing at the functions of desmin in modulating nuclear events. According to an earlier study by Li (1994), desmin displays significant similarity to the myogenic members of the helix-loop-helix (HLH) motif-containing family, particularly myoD, myogenin, and KE2-binding protein E12 (Murre et al., 1989; Li et al., 1994). Desmin also shows similarity to the basic and leucine zipper domains of jun, fos, and CREB transcription factors (Li et al., 1994). These similarities were associated with the functions of desmin in signal transduction and transport of myogenic factors to the nucleus or the modulation of chromatin conformation (Figure 1b). The study also shows that inhibition of desmin expression interferes with myoblast fusion and myotube formation. Moreover, desmin inhibition or reduction inhibits the expression of muscle-specific genes, namely myoD, myogenin, α-sarcomeric actin, and muscle creatine kinase (Li et al., 1994). Furthermore, mutations in desmin cause downregulation at the early expression of nkx2.5 and hamper the cardiomyogenesis (Höllrigl et al., 2002, 2007). All these data suggest that desmin could be a key molecule in the regulation of myogenesis as a nuclear element.

Another and the most curious partner in crime for desmin is the nuclear lamin. Lamins are bona fide nuclear proteins. They provide mechanical support to the nucleus, function in DNA repair, cell signaling, and transcription (Aebi et al., 1986; Liu et al., 2005; Manju et al., 2006; Gonzalez et al., 2008; Andrés and González, 2009; Malhas et al., 2009). There are two types of lamins (lamin A/C and lamin B) based on sequence homology. Lockard (1993) has shown that desmin and lamin B can be associated at the nuclear pore complex (NPC) in cardiac myocytes (Lockard and Bloom, 1993) and suggested that this trans-cellular desmin-lamin B network could be involved in directing the nucleo-sarcoplasmic exchange, influence the structure and function of DNA and so on (Lockard and Bloom, 1993). One can hypothesize from this observation that both lamin B and desmin are anchored to the NPC, which is how they interact (Figure 1b). With the fact that desmin has a lamin B binding domain, this hypothesis gets stronger (Georgatos et al., 1987). Considering earlier literature (Murre et al., 1989; Li et al., 1994; Höllrigl et al., 2002, 2007), a possible implication of the desmin-lamin B interaction might be the activation of myogenic HLH factors (Capetanaki et al., 1997). Our previous findings have shown that desmin and lamin B co-localize in the skeletal muscle tissue section from a healthy individual (no sign of muscle disease) but not in Limb-Girdle Muscular Dystrophy 2R (LGMD2R) patient who has severe muscle degeneration (Çetin et al., 2013). The LGMD2R phenotype is caused by a desmin mutation (c.1289-2A>G) and causes no alterations in desmin expression (Çetin et al., 2013); however, further studies have shown that the response to the mechanical loading was decreased in the patient (Ünsal, 2019). From this observation, we postulated that the loss of co-localization hampers the mechanotransduction cascade in the patient. These results point out a critical role for desmin in mechanotransduction. 

To investigate for proof for this peculiar interaction, we have used zebrafish muscle tissue. Co-immunoprecipitation studies, coherent with the literature, showed a physical association between desmin and lamin B (Kural-Mangıt and Dinçer, 2021). Additional studies showed that desmin also co-precipitates with Nup214 (Kural, 2017), which supports assumptions from the earlier study by Lockard (1993) (Lockard and Bloom, 1993) (Figure 1b). Of special interest, to explore the binding partners for desmin and lamin B, and to understand the extent of this relationship, we have performed a mass spectrometry analysis. One of the interesting candidates as a binding partner for desmin is Nup153 (Figure 1b). According to our data obtained from Co-IP experiments and proteomic analysis, desmin anchoring to NPC (Lockard and Bloom, 1993) can occur via Nup214 and Nup153 (Figure 1b). Furthermore, Nup153 also associates with lamin B via its C-terminal domain (Al-Haboubi et al., 2011). All these data support the ‘’interaction at NPC’’ hypothesis by Lockard (1993) (Lockard and Bloom, 1993).

Another interesting partner for desmin is a histone methyltransferase protein: SET and MYND domain-containing 1a (smyd1a). In zebrafish, smyd1a localizes in the nucleus and is required for myofiber maturation and muscle contraction (Tan et al., 2006). Considering the functions, we postulate that these two proteins, desmin and smyd1a, might be involved in the development or differentiation of skeletal muscle tissue. 

Tropomodulin (tmod4) is another candidate protein partner for desmin and the desmin-lamin B network. This actin minus-end protein has an NLS and possible function in the proliferation and differentiation of muscle cells (Kong and Kedes, 2004). It may seem that lack of hard evidence of interaction in literature lowers the probability of tmod4 being a part of the desmin-lamin B network; however, considering its functions in muscle cells, it would be interesting to think desmin-tmod4 interaction might somehow take part in the regulation of muscle development processes. 

The final potential interactor of desmin acquired from our results is the phosphoglycerate mutase (pgam2), a glycolytic enzyme, which has critical effects on muscle fusion and development (Qiu et al., 2008; Tixier et al., 2013). Pgam2 has been shown to localize in the nucleus (Qiu et al., 2008). Furthermore, the study by Tixier (2013) on zebrafish shows knockdown of pgam2 causes thin muscle phenotype, and these results suggest a role for glycolysis on muscle growth based on myoblast fusion (Tixier et al., 2013). From these, we postulate that desmin and pgam2 might involve in muscle development. 

These postulations and assumptions must be tested in a wet lab before jumping to any conclusion. Yet, it is still interesting to imagine the spectrum of different functions that desmin can undertake in the nucleus.

## 4. Protein localization, transport, and diseases

Mutations in the desmin gene causes skeletal and cardiac myopathies known as desminopathies. There is not a treatment for desminopathies thus far (Langer et al., 2020). The pathology caused by desmin mutations usually emerge from dysfunctional desmin network due to the desmin aggregation or myofibrillar degeneration, or the mutation interferes with PTMs or protein-protein interaction sites (Capetanaki et al., 2015). More than 70 mutations in the desmin gene have been associated with desminopathies (Capetanaki et al., 2015), and six of them (Ser12Phe, Ser13Phe, Arg16Cys, Ala285Val, Ser298Leu, Asp312Asn) are located within the NLSs on desmin (Szeverenyi et al., 2008) (Figure 2). These mutations are related to desmin aggregation (Ser12Phe, Ser13Phe, Ala285Val, Ser298Leu, Asp312Asn) (Bergman et al., 2007; Taylor et al., 2007; van Tintelen et al., 2009; Hong et al., 2011; Tse et al., 2013; Brodehl et al., 2018) and defects in network formation (Ser13Phe,Arg16Cys) (Pica et al., 2008; Sharma et al., 2009). Ser12Phe and Ser13Phe mutations also overlap with the phosphorylation sites on desmin (Figure 2). It is postulated that the Ser12Phe and Ser13Phe mutations might affect desmin’s phosphorylation status and interfere with filament polymerization and depolymerization (Pica et al., 2008; Hong et al., 2011). However, none of these studies have focused on how (or if) desmin transport might affected by these mutations. We believe there are two main reasons why the ‘how and if’ questions were not investigated: First, the researchers were focused on the cause of disease pathology and not the yet-undiscovered nuclear function of desmin, and, secondly, since the primary pathology of the disease is right on the table, no need arises for a detailed further investigation. For desminopathies, there is an experimental study aim to reduce desmin aggregation (Cabet et al., 2015). Nonetheless, it is not clear how the information obtained from this study can be translated into the treatment of desminopathies, and these studies usually did not focus on the subcellular localization of desmin since the ‘’nuclear desmin’’ concept is relatively new.

Besides the potential to understand the structure of NPC and transport mechanisms and function of a protein better, the cellular localization and how it is regulated might also reveal the targetability of a protein. The precise localization of a protein can control the accessibility of the interaction partners and molecules that regulate PTMs and allows the protein to integrate into the biological networks in the cell. Apart from causing aggregation and defects in filament formation, the mutations within the desmin NLSs and NES can alter the subcellular localization of desmin by blocking PTM sites and preventing desmin from entering the nucleus. It has been known for a long time that fault in subcellular localization or transport of a protein may result in diseases related to protein aggregation, biosynthesis, or cell metabolism (Kaiser et al., 2004; Sabherwal et al., 2004; Mendes et al., 2005; Mizutani et al., 2007; McLane and Corbett, 2009; Hoover et al., 2010; Shoubridge et al., 2010; Hung and Link, 2011). Hence, the clarification of the mechanism of transport of a protein has become a very attention-grabbing area. Understanding the transport not only indicates the controllability of protein activity but also allows revealing possible pathways associated with the biological processes of interest.

One other benefit that might arise from localization and transport studies is the broadening of our understanding of NLSs and NESs. There is a growing body of research on increasing the therapeutic targetability of the nucleus. For example, some researchers use modified NLSs to increase the efficiency of nuclear transport (Wilson et al., 1999; Escriou et al., 2003) and utilize the NLS characterization studies to understand the effects of modifications on delivery efficiency. Another therapeutic approach is based on the inhibition of nucleocytoplasmic transport mechanisms. There are many different and successful studies, especially in cancer research (Mahipal and Malafa, 2016; Kim et al., 2017). These studies clearly demonstrate the importance of the detailed analysis of basic biological processes.

## 5. Conclusions

The researchers studying the subcellular localization of proteins must ask: What is the biological significance of IF proteins in the nucleus? IFs act as a sensor and transmitter for extracellular signals in the cytoplasm, while nucleocytoplasmic transport of these proteins helps to regulate basal and adaptive cellular responses. The nucleocytoplasmic localization of the proteins that shuttle between cytoplasm and nucleus -shuttling proteins, changes perpetually to adapt to the extracellular environment. Thus, the subcellular localization of the shuttling proteins must be tightly controlled. Subcellular localization of the shuttling proteins can be affected by several factors such as interaction partners (for example, transport proteins) or the cellular state (proliferation, differentiation, etc.). This means that a change in the balance of the subcellular localization of the protein can cause either depletion or accumulation of the protein in the nucleus, which can result in impairment of the nuclear functions (Kumeta et al., 2012). All these suggest that the nuclear cytoskeletal proteins are as central for the nuclear responses as in the transduction of the signals from the plasma membrane. As for desmin, it is now known that desmin can occur and function inside the nucleus (Fuchs et al., 2016; Kural-Mangıt and Dinçer, 2021). Evidence on literature and our findings strongly suggest that desmin has a transcriptional regulatory role in the cell in addition to its cytosolic functions. Uncovering these functions, along with the binding partners and the network they generate, will contribute to the revelation of new roles of desmin in health and disease and how the nuclear transport may be involved and/or affected in facilitating highly orchestrated signaling processes.
